# Deep brain activation patterns involved in virtual gait without and with a doorway: An fMRI study

**DOI:** 10.1371/journal.pone.0223494

**Published:** 2019-10-21

**Authors:** Véronique Marchal, Jason Sellers, Mélanie Pélégrini-Issac, Cécile Galléa, Eric Bertasi, Romain Valabrègue, Brian Lau, Pierre Leboucher, Eric Bardinet, Marie-Laure Welter, Carine Karachi

**Affiliations:** 1 Sorbonne Universités, UPMC Univ Paris, CNRS, INSERM, AP HP GH Pitié Salpêtrière, Institut du Cerveau et de la Moelle épinière (ICM), Paris, France; 2 Sorbonne Université, CNRS, INSERM, Laboratoire d’Imagerie Biomédicale, LIB, Paris, France; 3 Centre de Neuroimagerie de recherche (CENIR), ICM, Paris, France; 4 Plateforme PRISME, ICM, Paris, France; 5 Service de Neurophysiologie, CHU Rouen, Université de Rouen, Rouen, France; 6 Service de Neurochirurgie, AP-HP, GH Pitié-Salpêtrière, Paris, France; Universidade Estadual Paulista Julio de Mesquita Filho, BRAZIL

## Abstract

The human gait program involves many brain areas such as motor cortices, cerebellum, basal ganglia, brainstem, and spinal cord. The mesencephalic locomotor region (MLR), which contains the pedunculopontine (PPN) and cuneiform (CN) nuclei, is thought to be one of the key supraspinal gait generators. In daily life activities, gait primarily occurs in complex conditions, such as through narrow spaces, or while changing direction or performing motor or cognitive tasks. Here, we aim to explore the activity of these subcortical brain areas while walking through narrow spaces, using functional MRI in healthy volunteers and designing a virtual reality task mimicking walking down a hallway, without and with an open doorway to walk through. As a control, we used a virtual moving walkway in the same environment. Twenty healthy volunteers were scanned. Fifteen subjects were selected for second level analysis based on their ability to activate motor cortices. Using the contrast Gait versus Walkway, we found activated clusters in motor cortices, cerebellum, red nucleus, thalamus, and the left MLR including the CN and PPN. Using the contrast Gait with Doorway versus Walkway with Doorway, we found activated clusters in motor cortices, left putamen, left internal pallidum, left substantia nigra, right subthalamic area, and bilateral MLR involving the CN and PPN. Our results suggest that unobstructed gait involves a motor network including the PPN whereas gait through a narrow space requires the additional participation of basal ganglia and bilateral MLR, which may encode environmental cues to adapt locomotion.

## Introduction

Though human gait is highly automated, the action of walking necessitates moving forward while constantly adapting the locomotor pattern to meet environmental constraints. In activities of daily living, an individual regularly walks in narrow spaces, passing through doorways, and frequently has to change gait trajectories and make half-turns. Recent imaging studies have revealed complex cortical and subcortical brain networks during real or imagined gait in healthy adults. During or just after real unobstructed gait, single photon emission computerized tomography (SPECT), positron emission tomography (PET), and near infrared spectroscopy (NIRS) studies have shown an activation of the premotor, primary sensorimotor, prefrontal, supplementary motor area (SMA), anterior cingulate, parahippocampal, fusiform and lingual gyri, precuneus and cuneus, superior parietal, and visual cortices, the thalamus, and the cerebellar vermis with extension to the brainstem within the mesencephalic locomotor region (MLR) [1,2]. The MLR contains the pedunculopontine (PPN) and cuneiform (CN) nuclei and is thought to be one of the key supraspinal gait generators [3–6]. During mental imagery of simple or unobstructed gait in healthy subjects, functional magnetic resonance imaging has shown similar brain area activations including SMA, parahippocampal, fusiform and lingual gyri, precuneus and cuneus, posterior cingulate, and visual cortices, putamen, subthalamic nucleus, MLR and cerebellar vermis and cortex activation, with decreased activity in the vestibular and somatosensory cortices [7–14]. Recently, resting state functional MRI connectivity of the MLR and cerebellar locomotor region was found to be related to gait capacity [15], and activation of the left PPN of the MLR to the speed of imaginary gait [10]. Very few studies have examined mental imagery of complex gait. In comparison to simple or unobstructed gait, imagined obstacle avoidance has induced a higher activation in middle occipital gyrus, middle frontal gyrus, and cerebellum [16], and navigation induced a higher activation in middle occipital gyrus and precuneus. Interestingly, in patients with Parkinson’s disease (PD) such complex gait circumstances, like walking through narrow spaces, half-turn or walking while performing another motor or cognitive task, could induce a sudden transient arrest of the course of gait, the so-called freezing of gait phenomenon [17]. In these PD patients with freezing of gait, dysfunction of the SMA-basal ganglia and SMA-PPN networks [18] during mental imagery of unobstructed gait, and altered cerebellar, sensorimotor cortices, basal ganglia, and inter-hemispheric resting-state connectivity have been reported [19–32], with an increased activation during complex gait relative to controls, thought to reflect a compensatory attempt to overcome altered neuronal activation [16]. These data suggest that cortico-basal ganglia-MLR networks are involved differently in simple (or unobstructed) *versus* complex walking.

Finally, the identification of the different brain networks involved in complex gait, such as walking through narrow spaces, represents a step further toward understanding how the human brain deals with walking in ecological conditions, and can be used to study dysfunction in pathological conditions. Here, we further explore the cortico-basal ganglia-MLR regions involved in complex gait in healthy volunteers by using a virtual reality task mimicking walking through a doorway from a first-person perspective in conjunction with functional MRI and validated 3D atlases [33]. As a control, we used a virtual moving walkway in the same environment. Here, we hypothesized that virtual gait through a doorway would induce an increased activation in the basal ganglia, in particular the subthalamic area known to be involved in the ability to switch to a more controlled (loco)motor behavior [34], and in the MLR, thought to be implicated in the ability to adapt locomotion in relation to attentional demands [35].

## Material and methods

### Subjects

Twenty right-handed healthy volunteers (age 29 ± 7 years; 8 males) participated in the fMRI study. The trial was supported by the Institut National de la Santé et de la Recherche Médicale (INSERM, C08-07, N°IDRCD/ 2008-A00324-51, ClinialTrial.gov Registration NCT02055261) and received approval from the local ethical committee of Paris 6-Pitié-Salpêtrière Hospital. All volunteers signed an informed consent in accordance with the Declaration of Helsinki.

All volunteers were familiarized to walk down a 10-meter-long hallway three times, at their natural speed, recorded using a stopwatch. For each volunteer, an average normalized gait speed (equal to gait speed/height) was calculated across trials.

### The virtual gait task

A virtual environment that closely mimicked the real hallway used for training was designed using 3D meshes with photorealistic textures and computer-generated lighting effects from 3D graphics software (Blender, The Blender Foundation, Amsterdam). Video game-like oscillations reproduced the pendular movement of the head while walking from a first-person perspective. These oscillations were absent for the control conditions.

**Fig 1:**
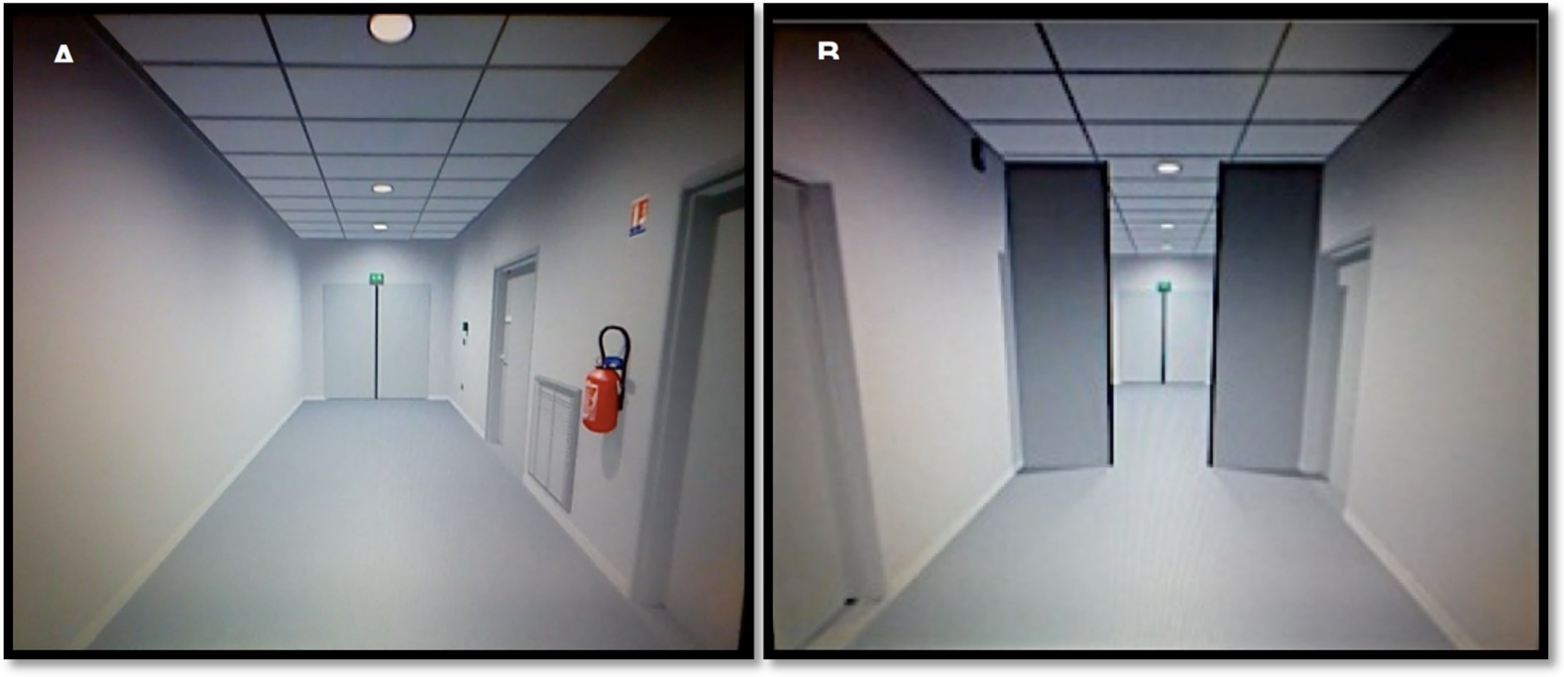
Screen captures of the virtual gait task. The task comprised pendular movements from a first person perspective to enhance imaginary gait during the fMRI session of gait without (A) and with (B) a doorway.

The virtual gait task consisted of four conditions. The first condition was imagining walking down the virtual hallway (Condition Gait, “GAIT”). The second condition consisted of imagining gliding smoothly on a moving walkway along the same hallway (Condition Walkway, “WALKWAY”). The third condition was imagining walking down the same hallway through a narrow open doorway (Condition Gait with Open Doorway, “GAIT DOORWAY”). The fourth condition consisted of imagining gliding smoothly on a moving walkway along the same hallway through the same narrow open doorway (Condition Walkway with Open Doorway, “WALKWAY DOORWAY”) (Fig 1). The position of the doorway within the hallway was randomized, with a minimal distance of 5 meters from the starting point, a maximal distance of 7 meters, and a doorway width of 83 centimeters [36].

The motion speed of the virtual environment was adapted to each subject’s average normalized natural gait speed for all four conditions. A PC running a 64-bit version of Windows 7 Professional Edition controlled stimulus presentation. The virtual reality environment was displayed using a video projector on a Plexiglas screen mounted on the rear end of the MR magnet bore. The displayed image covered 26.1° of the subject’s visual angle. Before entering the MR scanner, volunteers were trained to perform the virtual gait task on a computer screen with the instruction to fixate their gaze on the end of the virtual hallway.

During the fMRI session, the virtual gait task comprised four runs. Each run was composed of ten trials of each condition (GAIT, WALKWAY, GAIT DOORWAY, WALKWAY DOORWAY). Conditions were pseudo-randomized across trials, ensuring that all conditions were present and two conditions of the same type never followed each other. Each trial began by displaying words on the screen to indicate the condition. When volunteers felt ready, they pressed the button of an fMRI-compatible single-button ergonomic handheld device with their right thumb, which started the animation. At the end of each trial, the screen turned black indicating a 5-second rest period.

### Behavioral data acquisition

In the scanner, the time to start the trial between stimulus presentation and button press was recorded for every trial. The mean time to start trial for each volunteer was calculated by averaging the 40 trials for each run.

Following the fMRI session, volunteers filled out an in-house self-assessment questionnaire on a 10-point scale in order to assess their engagement, their feelings about the speed used, and their perception of the differences between conditions.

### Image acquisition protocol

Images were acquired with a 32-channel head coil on a Siemens 3T Verio scanner. T_2_*-weighted echo-planar images recording functional BOLD signals were acquired: volumes with 75 contiguous axial slices, interleaved acquisition, multi-band acceleration factor: 3, echo time (TE): 30 ms, repetition time (TR): 2.4 s, field of view (FOV): 20.5 cm x 20.5 cm, matrix: 114 x 114, voxel size: 1.8 x 1.8 x 1.8 mm. The FOV was carefully positioned to cover the whole brain including the brainstem. Functional images were acquired in the posterior to anterior phase. The number of volumes acquired during a trial depended on each volunteer’s time to perform the virtual gait trial (i.e. the elapsed time between the time to start and the time to complete the trial), also dependent upon the frame rate of the virtual environment (mean number of volumes 300±48). High-resolution T_1_-weighted anatomical images were acquired using an MPRAGE sequence: 176 sagittal slices, FOV: 25.6 cm x 25.6 cm, matrix: 256 x 256, TE: 4.18 ms, TR: 2.3 s, voxel size: 1.0 x 1.0 x 1.0 mm.

### Functional MRI data preprocessing

Images were preprocessed using the SPM8 statistical parametric mapping software (http://www.fil.ion.ucl.ac.uk/spm8) implemented in MATLAB® (The MathWorks, Inc.). Images were corrected for rigid body motion. Susceptibility artifacts were corrected by unwarping using TOPUP from the FSL suite (http://fsl.fmrib.ox.ac.uk/fsl/fslwiki/). Following co-registration of functional and anatomical images, the anatomical images were segmented and normalized to the Montreal Neurological Institute (MNI) standard space. The functional images were then normalized using similar parameters and smoothed with a 3-mm-full-width-at-half-maximum isotropic Gaussian kernel. The time series were high-pass filtered (cut-off period 128 seconds) to remove low-frequency noise. All images were processed with the SPM8 toolbox ArtRepair Despike to reduce motion artifact resulting from the multiband fMRI sequence (http://cibsr.stanford.edu/tools/human-brain-project/artrepair-software.html).

### Functional MRI data analysis

Statistical analyses were carried out using SPM8 to study the two main contrasts of interest: GAIT-WALKWAY to assess the positive effect of gait without doorway, and GAIT DOORWAY-WALKWAY DOORWAY to assess the positive effect of gait with doorway.

As our purpose was to examine deep brain structure activity during imagined walking *with versus without* doorway, we first performed a preliminary analysis to detect subjects that activated motor cortices, which would be expected with our virtual gait task, as reported previously during imagined or observation walking [10,14,37]. Individuals with a significant increase in BOLD signal for the global effect of gait using the (GAIT+GAIT DOORWAY)-(WALKWAY+WALWAY DOORWAY) contrast in a motor/premotor cortical mask of interest were then included in the final analysis. The mask chosen was designed using the Automated Anatomical Labeling atlas [38] and included the precentral gyrus (areas 1–2, which include the limbs representation of Brodmann areas 4 and 6) and the SMA (areas 19–20, corresponding to Brodmann area 6) bilaterally [8, 10].

For each volunteer, an individual block-design fixed-effect general linear model was designed that included the four conditions modeled on a trial-by-trial basis as square-wave functions convolved with the canonical hemodynamic response function of SPM8. Trials began when the subject pressed the button starting the animation and stopped when the virtual task was completed (i.e. when the subject reached the end of the hallway). Motion parameters for translation (x, y, z) and rotation (yaw, pitch, roll) were added to the GLM as covariates of non-interest. The parameters of the general linear model were estimated within an explicit mask of the subject’s brain (namely, the canonical brain mask from SPM8). Then, only volunteers showing significant activation (multiple comparisons corrected at the cluster level for p<0.05 using the false discovery rate procedure; cluster-defining threshold: p<0.001 uncorrected [39,40] for the global effect of gait within the motor/premotor cortical mask) were considered for the subsequent analyses. Based on this analysis, among the 20 volunteers, 15 were ultimately selected. We removed the data of 5 participants from the final analyses due to: 1) abrupt head movements larger than 2mm in two subjects, 2) a prefrontal hypersignal during the recording of the EPI sequence in another subject, possibly due to a technical problem of the coil channels and 3) no activation in the primary motor cortex of the lower limb in the two remaining subjects (p<0.001 uncorrected for multiple comparisons at the whole brain level).

An individual first-level event-related fixed-effect general linear model was then designed that included the four conditions modelled on a trial-by-trial basis as stick functions convolved with the canonical hemodynamic response function, time-locked to the button press marking the onset of animation. Rigid motion parameters were added to the general linear model as covariates of non-interest. The parameters of the general linear model were estimated within the same explicit canonical brain mask as previously. Contrast images were computed for the movement x doorway interaction ([GAIT-WALKWAY]-[GAIT DOORWAY-WALKWAY DOORWAY]) and the two contrasts of interest (GAIT-WALKWAY and GAIT DOORWAY-WALKWAY DOORWAY), smoothed with a 3-mm-full-width-at-half-maximum isotropic Gaussian kernel, and entered into separate second-level random-effect group analyses. Finally, one-sample t-tests were carried out for group statistical inference, and multiple tests were false discovery rate-corrected at the cluster level for p<0.05 (cluster-defining threshold: p<0.001 uncorrected).

To assess deep brain structure activations, inference was further limited to a large region of interest including the basal ganglia and the brainstem. This region of interest was obtained automatically by registering the YeB atlas [33] to the MNI152 anatomical template, converting all structures of the atlas to binary masks, and using morphological operations (dilations and closings) to yield a single almost convex binary region of interest (volume: 125 cm^3^).

### Anatomical location of activated clusters

Cortical and cerebellar activated clusters were anatomically labeled by overlaying the thresholded activation maps onto Brodmann and Automated Anatomical Labeling atlases using MRIcron software (http://www.nitrc.org/projects/mricron/). For the thalamus, basal ganglia and brainstem, the YeB atlas registered to the MNI152 anatomical template (1 mm isotropic) was used to localize activated clusters [10].

## Results

### Behavioral results

The mean time to start of the 15 volunteers for the virtual gait task was 3096 ± 281 ms, with no significant difference in the mean time between conditions (GAIT, WALKWAY, GAIT DOORWAY, WALKWAY DOORWAY) and volunteers (one-way ANOVA, F (3.52) = 0.18, p = 0.91). The mean normalized virtual speed across volunteers was 0.88 ± 0.06 s^-1^. Using our in-house self-assessment questionnaire, the 15 volunteers scaled their ability to stay focused during the task at 6/10 (mean, σ = 1.25), their feeling of walking at 6.7/10 (mean, σ = 1.33), and their ability to distinguish between gait and walkway conditions at 6.9/10 (mean, σ = 2.2). The motion speed of the virtual environment was well adapted for 13 of the 15 volunteers.

### Functional MRI results

Activated clusters found at cortical and subcortical levels for the gait *versus* walkway conditions, both with and without doorway are listed in Table 1.

**Table 1:** Activated clusters found in the brain regions for the contrasts GAIT-WALKWAY and GAIT DOORWAY-WALKWAY DOORWAY.

ACTIVATED CLUSTER	CLUSTER SIZE (Voxels)	MNI coordinates (x,y,z in mm)	z-score	BRAIN AREAS (Brodmann Areas)
**GAIT-WALKWAY CORTICAL AND CEREBELLAR**				
CL-1	2170	-15,-18,74	4.00	R and L Precentral gyrus (BA 4–6)
		6,-8,57	3.96	R and L SMA (BA 6)
CL-2	4786	3,-97,6	4.06	R Occipital Lobe (BA 17–18)
		0,-38,-20	5.28	R Cerebellum I-IV-V L Cerebellum I-IV-V-VI Cerebellum (Vermis VI)
**GAIT-WALKWAY SUBCORTICAL AND MLR**				
CL-3	140	-3,-28,3	3.64	R and L Thalamus (VIM-MD)
CL-4	54	-18,-22,12	3.37	L Thalamus (VIM-MD)
CL-5	4	-16,2,24	3.22	L Caudate
CL-6	43	10,6,-4	3.75	R Caudate R Accumbens Nucleus
CL-7	5	-21,2,-3	3.15	L Putamen
CL-8	4	-27,2,6	3.14	
CL9	15	18,0,-9	3.34	R Pallidum (GPe)
CL-10	16	9,-22,-6	3.36	R Red Nucleus
CL-11	29	-2,-31,-14	3.48	L CN
CL-12	37	-8,-24,-16	3.51	L PPN
CL-13	33	-14,-26,-9	3.29	L Substantia Nigra
**GAIT DOORWAY-WALKWAY DOORWAY CORTICAL AND CEREBELLAR ACTIVATED CLUSTERS**				
CL-a	1143	-16,-18,70	3.50	L Precentral gyrus (BA 4–6)
		-9,-19,62	3.78	R and L SMA (BA6)
**GAIT DOORWAY-WALKWAY DOORWAY SUBCORTICAL AND MLR ACTIVATED CLUSTERS**				
CL-b	227	-18,14,4	3.34	L Caudate L Putamen
		-24,5,4	3.43	
CL-c	22	10,8,0	3.32	R Caudate R Accumbens
CL-d	15	-12,-2,-6	3.26	L Internal Globus Pallidus
CL-e	4	12,-8,-6	3.21	R Subthalamic nucleus
CL-f	104	-8,-24,-14	3.67	L CN L PPN L Substantia Nigra
CL-g	11	8,-28,-8	3.40	R CN R PPN

Contrasts for GAIT-WALKWAY are numbered 1 to 13, and for GAIT DOORWAY-WALKWAY DOORWAY a to g.

BA = Brodmann area; L = left; R = right. Inference false discovery rate-corrected at the cluster level for p<0.05 (cluster-defining threshold: p<0.001 uncorrected, critical cluster extent False discovery rate-corrected = 2170 for GAIT-WALKWAY and extent False discovery rate-corrected = 1143 for GAIT DOORWAY-WALKWAY DOORWAY.

### Effect of gait (GAIT-WALKWAY)

**Fig 2:**
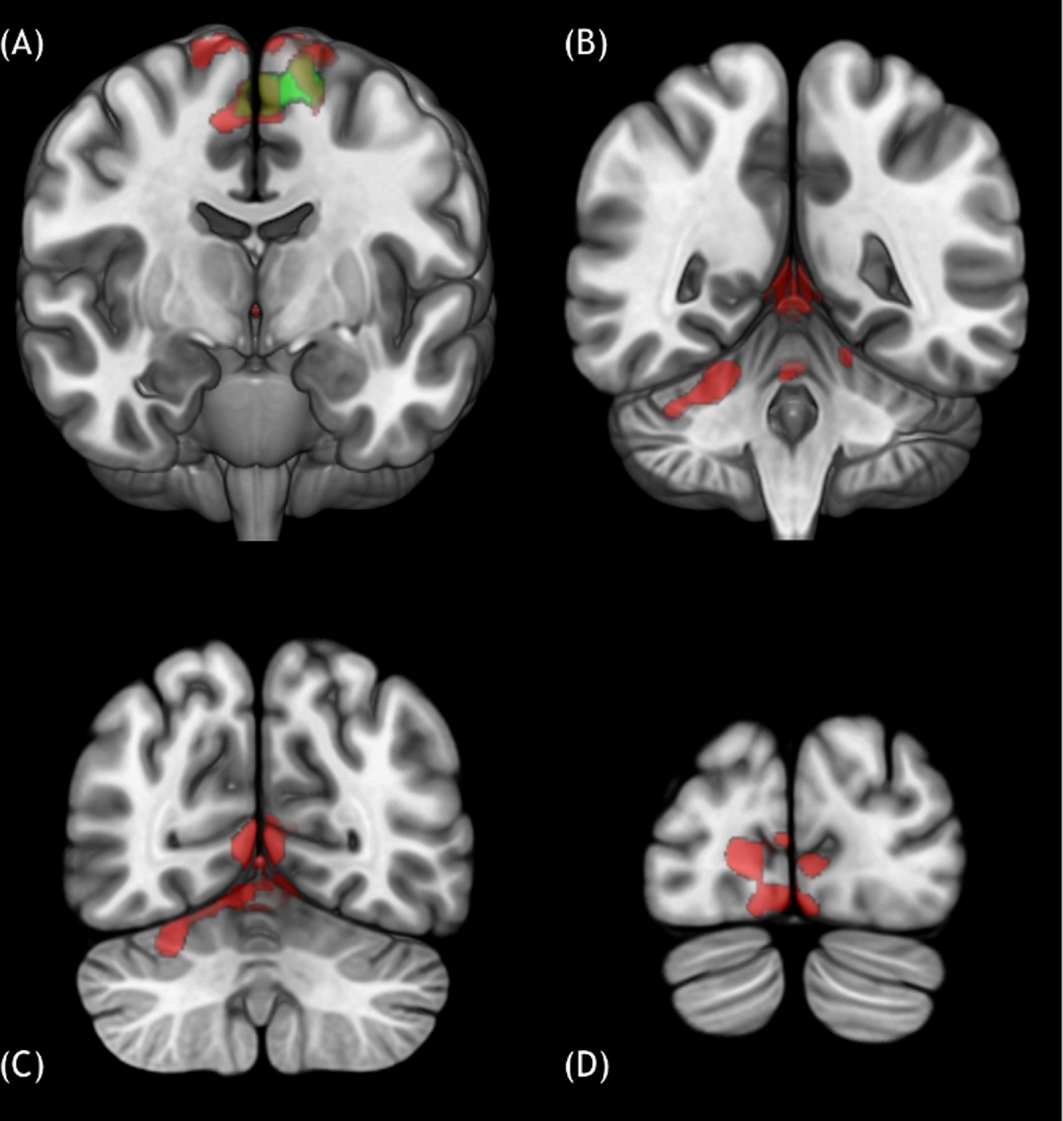
Cortical and cerebellar activated clusters for the two contrasts of interest ‘GAIT–WALKWAY’ and ‘GAIT DOORWAY–WALKWAY DOORWAY’. The contrasts are represented in the MNI152 template space. A, B, C, D: three-dimensional views of the MNI152 template in frontal cross-sections with activated clusters. GAIT-WALKWAY (red) showed clusters in the bilateral motor cortices (A) including the SMA, premotor and motor cortices (BA 4–6), and the cerebellum (left lobe VI, bilaterally in the lobes I-IV, V, and in the vermis VI) (B, C). Activations were also found in the right occipital lobe (BA 17–18) (D). GAIT DOORWAY-WALKWAY DOORWAY (green) showed activated clusters only in the SMA, premotor and motor cortices (BA 4–6) (A).

In addition to bilateral activations of SMA and precentral gyrus (corresponding to motor and premotor cortices of the limbs (BA 4 and 6), the positive effect of gait (GAIT-WALKWAY) showed two activated clusters (Fig 2). One included different cerebellar areas (left hemisphere VI; bilateral hemisphere I, IV, V; vermis VI) and the right occipital lobe (BA 17–18) (Fig 2).

**Fig 3:**
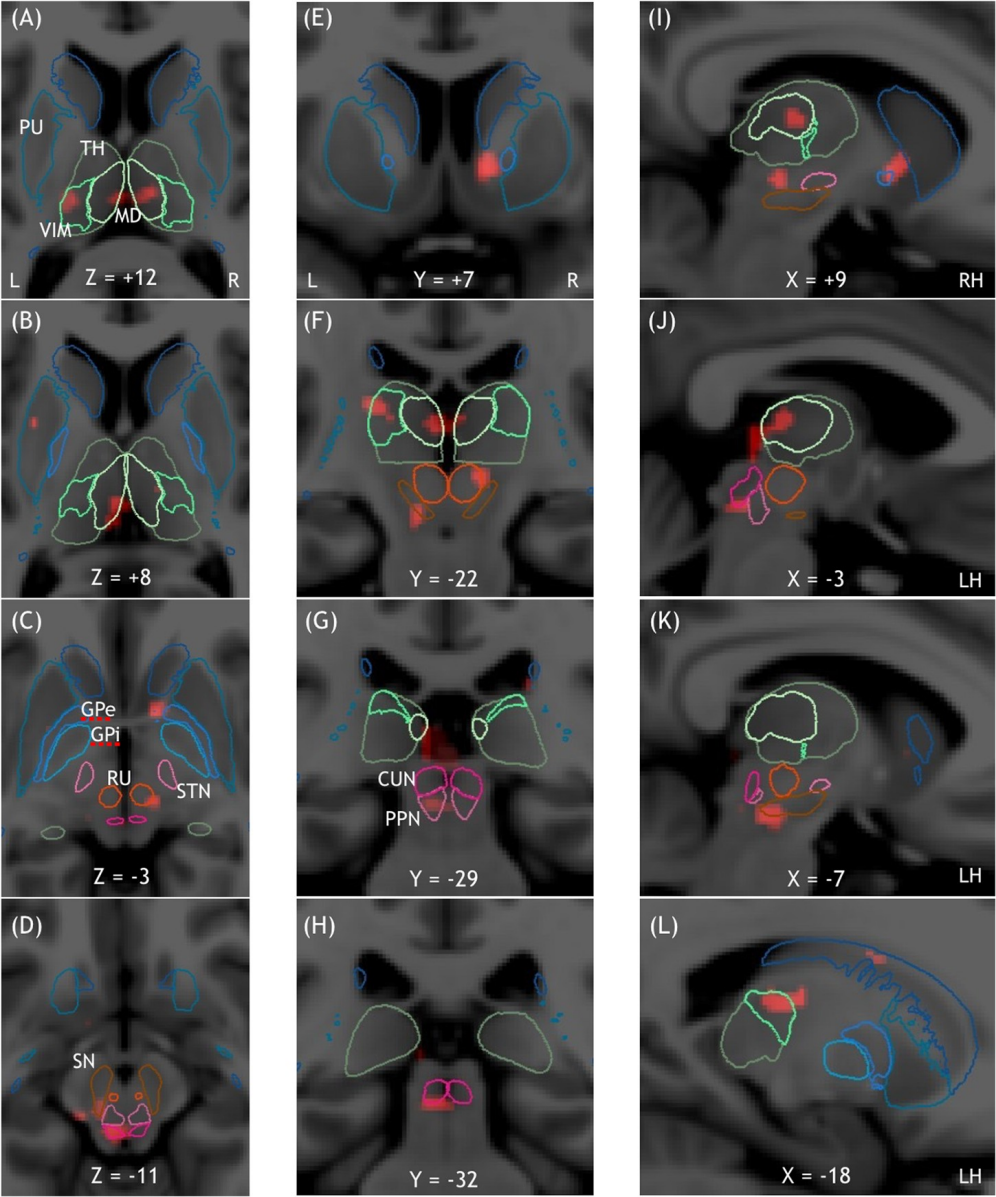
Midbrain clusters of the GAIT -WALKWAY contrast represented overlaid on the MNI152 template. The contours of the nuclei of the thalamus, the basal ganglia, and the MLR are given by the YeB atlas mapped to the MNI template. Midbrain and MLR clusters are shown in horizontal (A, B, C, D), frontal (E, F, G, H), and sagittal (I, J, K, L) sections. GAIT -WALKWAY showed clusters in the thalamus (VIM (left) and MD nuclei) (A, B, F, I, J, L), left caudate nucleus (L), left putamen (B), right red nucleus (C), left substantia nigra (D, K), and left CN and PPN (D, G, H, J).

We also found activated clusters in the basal ganglia with one cluster in the thalamus (left ventral intermediate median and bilateral medio-dorsal nuclei), two in the striatum (one involving the right head of the caudate and the right accumbens nuclei and a small one in the left body of the caudate nucleus), one in the right red nucleus, one in the left putamen, and one in the right external pallidum. In the MLR, we found three activated clusters: two located in the left PPN and one involving the ventral part of the left CN (Fig 3).

The negative effect of gait (WALKWAY-GAIT) showed no activated cortical, cerebellar, or subcortical clusters.

### Effect of gait with doorway (GAIT DOORWAY-WALKWAY DOORWAY)

We found no additional activated cortical or cerebellar clusters for GAIT DOORWAY-WALKWAY DOORWAY conditions, except activations of bilateral SMA and left precentral gyrus, corresponding to motor and premotor cortices of the limbs (BA 4 and 6) (Fig 2).

**Fig 4:**
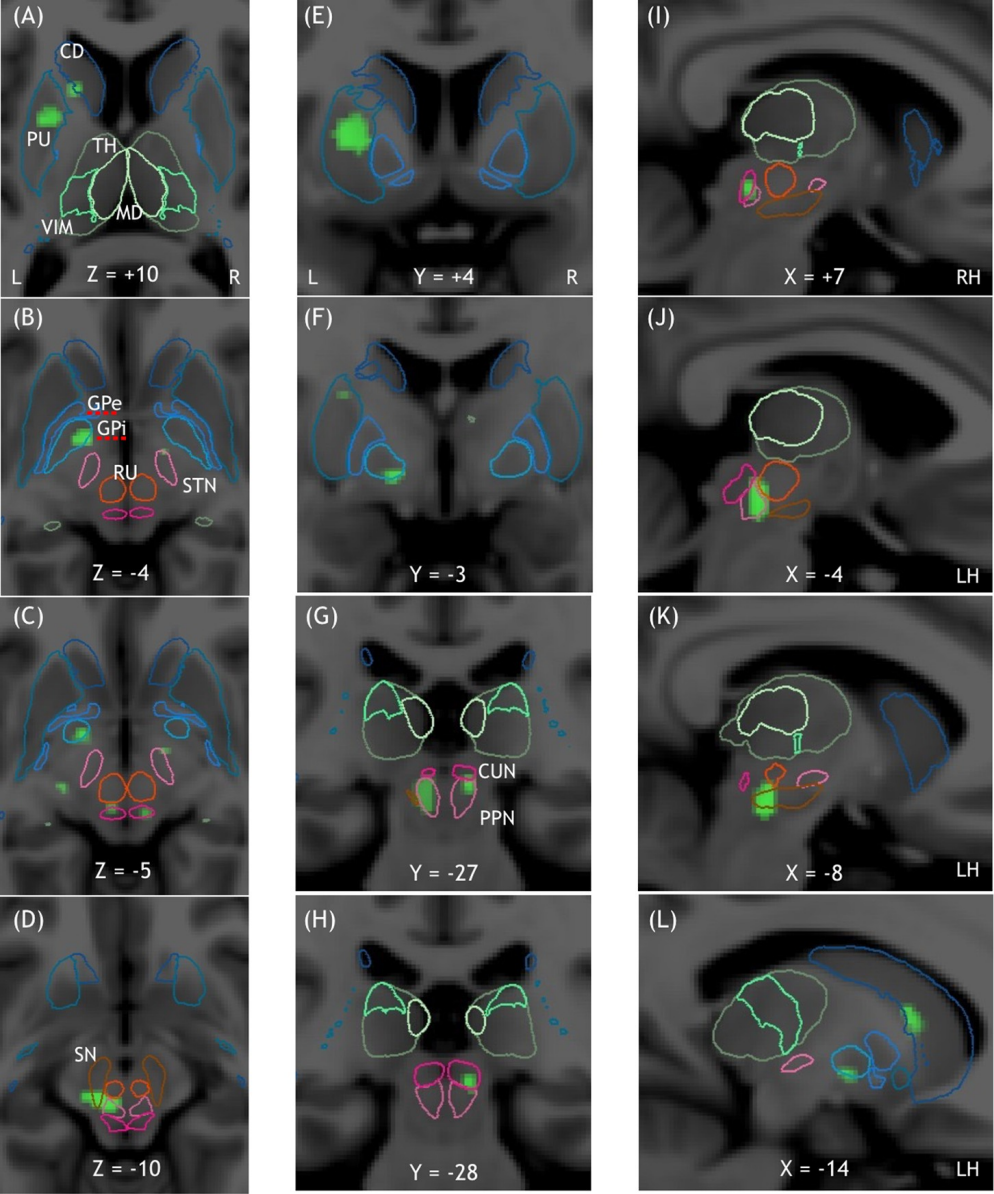
Midbrain clusters of the GAIT DOORWAY-WALKWAY DOORWAY contrast represented overlaid on the MNI152 template. The contours of the nuclei of the thalamus, the basal ganglia, and MLR are given by the YeB atlas mapped to the MNI template. Midbrain and MLR clusters are shown in horizontal (A, B, C, D), frontal (E, F, G, H), and sagittal (I, J, K, L) sections. GAIT DOORWAY—WALKWAY DOORWAY showed clusters in the left caudate nucleus (A, L), left putamen (A, E, F), left internal globus pallidus (B, C, F, L), right subthalamic nucleus (B, C), bilateral CN and PPN (D, G, I, J, H), and left substantia nigra (D, K).

In the basal ganglia, during GAIT DOORWAY-WALKWAY DOORWAY, we found four activated clusters: one in the left striatum including the tail and the head of the caudate nucleus and the putamen, one in the right caudate and accumbens nuclei, one in the left internal globus pallidus, and a small one involving the right subthalamic nucleus area. In the MLR, we found two activated clusters: one located mainly in the left PPN including the left CN extending towards the substantia nigra pars reticulata, and one smaller located in the right CN extending towards the dorsal part of the right PPN (Fig 4).

The negative effect of gait with doorway (WALKWAY DOORWAY-GAIT DOORWAY) showed no activated cortical, cerebellar, or subcortical clusters.

### Interaction effect (GAIT-WALKWAY)-(GAIT DOORWAY-WALKWAY DOORWAY)

We found no significant positive interaction effect for basal ganglia structures. In the MLR, we observed a trend for a positive interaction involving the left CN and PPN using a liberal uncorrected threshold of 0.15.

## Discussion

In this study, we created a virtual reality environment to reproduce walking through a doorway and identified activated subcortical brain structures during gait within this environment. In healthy subjects, we observed different activations during mental imagery of unobstructed gait and gait with doorway. During imagined unobstructed gait, we found activated clusters in motor cortices, cerebellum, red nucleus, thalamus, and the left MLR including the PPN. During imagined gait through a doorway, we found activated clusters in motor cortices, left putamen, left internal globus pallidus, left substantia nigra pars reticulata, right subthalamic nucleus area, and bilateral MLR involving the CN and PPN. Our results suggest that unobstructed gait involves a motor network including the PPN whereas gait through a narrow space requires the additional participation of basal ganglia structures and the bilateral MLR, which may encode environmental cues to adapt locomotion [41].

### Mental imagery of gait with doorways preferentially activates basal ganglia and mesencephalic locomotor region

In our subjects and with our task, mental imagery of gait both with and without doorways induced an activation of subcortical regions previously reported to be activated during mental imagery of straight and unobstructed gait, i.e. the striatum (bilateral caudate nuclei, left putamen) and left MLR [8,42]. Conversely, only unobstructed gait without doorways significantly induced activations of the primary and secondary visual cortices, the right and left cerebellum hemispheres, and vermis of the cerebellum and its output structures, the ventral intermediate and median nuclei of the thalamus and the red nucleus. These brain areas are known to be involved in visuospatial navigation, motor coordination, and postural control during gait and standing position and have previously been shown to be activated during mental imagery of standing and straight-usual gait in healthy adults [9,42–44]. Interestingly, contrary to previous studies that have shown increased parietal and occipital activations during precision gait and gait with obstacles [7,45], we saw less activation of these cortices during gait through a narrow space. During gait through a doorway, we observed a preferential recruitment of basal ganglia and MLR nuclei, with less activity within the cerebellum-thalamic network. Indeed, we found activated clusters not only in the left sensorimotor territory of the putamen and the right subthalamic nucleus area, but also the internal globus pallidus and substantia nigra pars reticulata, the two main output structures of the basal ganglia. Together with these activations, we also found bilateral activations of the MLR including the CN and PPN. These recruited nuclei may encode environmental cues together in order to adapt posture and locomotion and may therefore play an integral role in the movements required for narrow space navigation. In pathological condition where gait may be interrupted while the subject attempts to walk through narrow spaces, such as freezing of gait in PD, increased functional connectivity of the SMA-MLR network and MLR activity have been reported during unobstructed gait mental imagery [18,46]. Freezing of gait episodes have also been related to dysfunction in executive control and impairment of conflict resolution in the setting of a loss of automaticity centers [47]. Finally, these data highlight the role of the basal ganglia and MLR, in connection with the SMA, in adapting the locomotor pattern in complex gait situations, and their probable dysfunction in pathological conditions that could affect the ability to freely walk in complex environments [16].

### Limitations

In our study we used a novel virtual reality task to enhance the experience of motor imagery and a control task using a virtual walkway to better contrast the gait condition while maintaining a similar visuospatial environment. Mental imagery has been shown to be reliable to activate locomotor networks in healthy volunteers [9,11] but mental ability can vary widely across subjects [48] To control mental imagery, subjects’ engagement and performance, we used both a self-assessment questionnaire and measured the time to start the trial, and only included in the final analysis the data obtained in participants based on their ability to recruit known motor and pre-motor brain centers involved in gait imagery. Even though we did not use a validated questionnaire for quantifying mental imagery ability of movement, such as the VMIQ-2 [13,49], we obtained positive scores for self-assessment of “feeling of walking” and “distinction between gait and walkway conditions” consistent with good ability of our subjects to imagine gait in a first-person perspective task. Previous studies report a significant relationship between chronometric measures of motor imagery and successful motor imagery [50,51]. We did not find any correlation between the time to start the trial and cortical activations. Since our task relied on a virtual environment, timing of the task was controlled across all volunteers and chronometric measures could therefore not be recorded. Ultimately we decided to select volunteers based on their ability to recruit bilateral cortical activations in the SMA and primary motor cortex during the task, this network including M1 (BA 4), SMA, and pre-SMA, which have been shown to be more strongly activated with mental imagery [7,52] and in good imagers [37]. Finally, even though we selected subjects according to the activations of these cortical areas, our primary interest was to characterize the role of deep brain structures during virtual gait for which we carried out an independent analysis. We did not find any significant positive interaction surviving a corrected threshold for interaction between gait and the presence of doorways but only a tendency at a liberal uncorrected threshold in the left CN and the left PPN. This suggests that such interaction actually could exist but may not be significant due to lack of power, likely attributable to small sample size and design complexity.

## Conclusion

Unobstructed gait in healthy volunteers involves a network including motor and visual cortical areas, cerebellum and related structures, and MLR, whereas gait through narrow spaces necessitates a larger deep brain network involving basal ganglia and MLR. Further studies are now needed to explore the dysfunction of these brain structures in PD patients with freezing of gait caused by various complex gait circumstances, such as walking through doorways.
